# How Well Do Core Faculty Understand The Emergency Medicine Milestones?

**DOI:** 10.5811/westjem.2019.11.44289

**Published:** 2019-12-19

**Authors:** Randy Sorge, Simiao Li-Sauerwine, Jorge Fernandez, Gene Hern

**Affiliations:** * Louisiana State University Health Sciences Center in New Orleans, Department of Emergency Medicine, New Orleans, Louisiana; † The Ohio State University, Department of Emergency Medicine, Columbus, Ohio; ‡ University of California San Diego School of Medicine, Department of Emergency Medicine, San Diego, California; § Highland Hospital – Alameda Health System, University of California San Francisco, Department of Emergency Medicine, San Francisco, California

## Abstract

**Introduction:**

It is unclear how emergency medicine (EM) programs educate core faculty about the use of milestones in competency-based evaluations. We conducted a national survey to profile how programs educate core faculty regarding their use and to assess core faculty’s understanding of the milestones.

**Methods:**

Our survey tool was distributed over six months in 2017 via the Council of Emergency Medicine Residency Directors (CORD) listserv. Responses, which were de-identified, were solicited from program directors (PDs), assistant/associate program directors (APDs), and core faculty. A single response from a program was considered sufficient.

**Results:**

Our survey had a 69.7% response rate (n=140/201). 62.9% of programs reported educating core faculty about the EM Milestones via the distribution of physical or electronic media. Although 82.6% of respondents indicated that it was important for core faculty to understand how the EM Milestones are used in competency-based evaluations, respondents estimated that 48.6% of core faculty possess “fair or poor” understanding of the milestones. Furthermore, only 50.7% of respondents felt that the EM Milestones were a valuable tool.

**Conclusion:**

These data suggest there is sub-optimal understanding of the EM Milestones among core faculty and disagreement as to whether the milestones are a valuable tool.

## INTRODUCTION

The emergency medicine (EM) Milestone Project was created in 2012 by the Accreditation Council for Graduate Medical Education (ACGME) and the American Board of Emergency Medicine as a standardized framework for the assessment of EM residents.^1^ The EM Milestones were developed through expert consensus and comprehensive literature review, as a result of the desire to move from a process-oriented to an outcomes-oriented focus, while retaining the six ACGME core competencies (patient care, medical knowledge, interpersonal communication, professionalism, practice-based learning and improvement, and systems-based practice).^2^ Outcome measurements were assigned to each of these core competencies and were intended to serve as a framework for residency curricula and individual evaluation. ACGME Milestones allow programs to assess for gaps in curricula and to monitor resident progress, including the potential need for remediation.^3^ Residency programs are required to evaluate their residents using milestones and to submit these assessments to the ACGME semi-annually.

It is unclear, however, how programs educate core faculty about the EM Milestones Project and if core faculty possess adequate understanding of the milestones in order to make accurate assessments. Finally, it is unknown whether PDs and APDs, who implement milestones measurements based on ACGME requirements, feel that milestones are a valuable tool to assess resident learning.

## METHODS

Our survey tool, which was designed as part of the Medical Education Research Certificate Program and deemed exempt by the Institutional Review Board at Alameda Health System (Highland Hospital), was comprised of 12 questions, 11 of which were multiple choice and one of which was free response (Appendix). To ensure face validity, the survey was piloted by six APDs at three authors’ home institutions prior to distribution. Feedback from the pilot resulted in minor changes to improve clarity, which were incorporated into the final survey. The survey was then distributed over a six-month period from July 2017 to January 2018 via the Council of Emergency Medicine Residency Directors (CORD) listserv. Responses, which were de-identified with respect to program, were solicited from program directors (PD), assistant/associate program directors (APD), and core faculty. A single response from a program was considered sufficient. Duplicate responses were reconciled by computer algorithm, prioritizing the responses of PDs over APDs over core faculty.

Respondents were asked about how they educate core faculty about the EM Milestones and to estimate their perceived understanding of the milestones on a 5-point Likert-type scale, where 1 = “no understanding,” 2 = “poor understanding,” 3 = “fair understanding,” 4 = “good understanding,” and 5 = “very good understanding.” Data were compiled and analyzed using Microsoft Excel.

## RESULTS

Of the 201 EM programs contacted, 144 responses were received, representing 140 unique programs (response rate 69.7%). The four duplicate responses were reconciled by computer algorithm, prioritizing the response of PDs over APDs over core faculty. 70.7% of responses were from PDs, 26.4% were from APDs, and 2.9% were from core faculty. 62.9% of programs reported educating core faculty about the EM Milestones via the distribution of physical or electronic media. Although 82.6% of respondents indicated that it was important for core faculty to understand how the EM Milestones are used in competency-based evaluations, respondents estimated that 48.6% of core faculty possess “fair or poor” understanding of the milestones (Table 1). Furthermore only 50.7% of respondents felt that the EM Milestones were a valuable tool.

## DISCUSSION

These data suggest that PDs and APDs perceive that there is suboptimal understanding of the EM Milestones amongst core faculty, which may stem from insufficient or inadequate faculty development in this area. If core faculty do in fact have a poor understanding of the milestones, it calls into question the validity of their evaluations. Further investigation may be warranted to determine the accuracy of these perceptions and to suggest recommendations to improve core faculty understanding.

**Population Health Research Capsule**

What do we already know about this issue?

*Core faculty are responsible for evaluating residents semi-annually based on the ACGME milestones*.

What was the research question?

How well do Core Faculty understand the milestones?

What was the major finding of the study?

*Nearly half of core faculty are felt to have a “fair or poor” understanding of the milestones*.

How does this improve population health?

*These findings suggest that there is room for improvement in terms of core faculty development in regards to the milestones*.

There also appears to be disagreement about the importance and value of EM Milestones. General themes in free-text comments included the following: that the EM Milestones were good in theory yet administratively burdensome in practice, that they tend to be more useful with regard to the remediation of struggling residents but not as valuable in evaluating the majority of well-performing residents, and that they could be at times counterproductive due to variable faculty interpretation of each sub-competency and what actually constitutes meaningful achievement of proficiency within each sub-competency.

This study highlights that there is still significant room for improvement in terms of core faculty development regarding EM Milestones and their current role in competency-based assessment.

## LIMITATIONS

The main limitation of this study is that the survey tool is subject to recall, sample, and response bias.^4^ Responders may be hesitant to answer truthfully to the questions out of fear of disparaging their own program. Another limitation is that responses were solicited from a representative sample of PDs, APDs and core faculty rather than directly from core faculty. A direct sampling was determined to be impractical due to the large number of responses required in order to draw meaningful conclusions. Therefore, the authors chose to solicit the perceptions of PDs and ADPs as a surrogate marker.

Efforts were made in this study’s design to reduce potential bias, including the development of a high-quality, brief, questionnaire. Pilot testing of the survey tool occurred with APDs at each of the authors’ programs in order to examine the quality and clarity of questions, ease of administration, potential for response fatigue, and to gather general feedback.

## CONCLUSION

The results of this survey demonstrate that there is variability in how EM programs educate core faculty about the EM Milestones. Furthermore, nearly half of respondents believe core faculty possess a “fair to poor” understanding of the EM Milestones. These results demonstrate an opportunity to improve faculty development with respect to the utility of milestones in competency-based assessment. Ultimately, this study identifies areas of need with respect to better educating educators themselves of the criteria by which the acquisition of knowledge, skills, attitudes, and behaviors is assessed during residency.

## Supplementary Information



## Figures and Tables

**Table 1:** Respondents’ perceived understanding of the emergency medicine (EM) Milestone Project by core faculty.

	Core faculty
Very good understanding	10.7%
Good understanding	40.7%
Fair understanding	35%
Poor understanding	13.6%
No understanding	0%
