# Supplementary material for: How Well Do Core Faculty Understand The Emergency Medicine Milestones?
Source: West J Emerg Med. 2019 Dec 19;21(1):160–2. doi: 10.5811/westjem.2019.11.44289 (PMC6948701; doi:10.5811/westjem.2019.11.44289)
Supplement: Supplementary file 1 [file wjem-21-160-s001.docx]

**APPENDIX:** Survey Instrument

| 1. Which residency program do you represent? 2. What is your current role at your residency program?    1. Programs Director    2. Associate/Assistant Program Director    3. Core Faculty 3. How do you educate your interns about the EM Milestone Project?    1. Lectures/didactics    2. Small group discussions    3. Individual meetings    4. Distributions of physical or electronic documents (e.g. residency handbook, policy manual, email, etc.)    5. None of the above 4. When do you first education your interns about the EM Milestone Project?    1. Prior to the start of residency    2. In the first month of residency    3. Within the first three months of residency    4. Within the first six months of residency    5. After six months into residency    6. We do not provide any education about the EM Milestone Project 5. Regarding the competency-based evaluations for interns, in which types of evaluations do you utilize the EM Milestones?    1. Semi-annual evaluations    2. Shift evaluations    3. Procedural evaluations    4. Simulation evaluations    5. End of rotation evaluations    6. We do not utilize the EM Milestones in our evaluations 6. In your opinion how well do interns understand the EM Milestones Project at the point of their first mid-year review?    1. They have a very good understanding    2. They have a good understanding    3. They have a fair understanding    4. They have a poor understanding    5. They have no understanding 7. How are core faculty at your residency program educated about the EM Milestones Project?    1. Lectures/didactics    2. Small group discussions    3. Individual meetings    4. Distributions of physical or electronic documents (e.g. residency handbook, policy manual, email, etc.)    5. None of the above 8. In your opinion, how well do your core faculty understand the EM Milestone Project    1. They have a very good understanding    2. They have a good understanding    3. They have a fair understanding    4. They have a poor understanding    5. They have no understanding 9. In your opinion, is it important for core faculty to understand how the EM Milestones are utilized in competency-based evaluations?    1. Yes    2. No    3. Undecided/No opinion 10. In your opinion, is it important for interns to understand how the EM Milestones are utilized in their competency-based evaluations?     1. Yes     2. No     3. Undecided/No opinion 11. In your opinion, are the EM Milestones a valuable tool for evaluating your interns?     1. Yes     2. No     3. Undecided/No opinion   If you choose, you may make any other comments regarding the EM Milestones in the space below. |
| --- |
